# Exploring Mental Health Patient's Perceptions and Aspirations in Employment

**DOI:** 10.1192/bjo.2022.218

**Published:** 2022-06-20

**Authors:** Holly Melvin, Adeola Akinola

**Affiliations:** 1 University of Manchester, Manchester, United Kingdom; 2 Pennine Care NHS Foundation Trust, Manchester, United Kingdom

## Abstract

**Aims:**

To explore mental health in-patient's perceptions and aspirations in employment and to produce a lay document on employment

**Methods:**

Employment is beneficial, it improves mental health and betters social integration. Different interventions exist to support mental health patients into employment. However, patients experience many obstacles, including the characteristics of their condition and stigma.

Literature review was undertaken, using a search string on PubMed focusing on mental health and employment.

Developed and used a 17-question questionnaire exploring patients’ perceptions and aspirations in employment

Created lay document containing information on benefits of employment, employment rights and accessing employment.

**Results:**

100% of patients interviewed were interested in employment. 90.9% believed employment would improve their mental health and 100% believed it would improve their connection to their community. 100% felt there is a stigma around mental health problems which makes finding employment harder.

**Conclusion:**

The barriers mental health patients seeking employment face are varied and complex. Most wish to seek employment, and should be encouraged, as the health benefits are clear. Professionals need to acknowledge individual barriers patients face including different mental health characteristics, ethnicity, gender and personal circumstances and find a way to create a bespoke service tailored to patients’ needs in order to secure employment.

